# Prediction of methotrexate efficacy and adverse events in patients with juvenile idiopathic arthritis: a systematic literature review

**DOI:** 10.1186/1546-0096-12-S1-P127

**Published:** 2014-09-17

**Authors:** Pieter Van Dijkhuizen, Nico Wulffraat

**Affiliations:** 1 Paediatric Immunology, Istituto Giannina Gaslini, Genova, Italy; 2 Paediatric Immunology, University Medical Centre Utrecht, Wilhelmina Children's hospital, Utrecht, Netherlands

## Introduction

Methotrexate (MTX) is the cornerstone disease-modifying anti-rheumatic drug in juvenile idiopathic arthritis (JIA). In JIA, it is important to start effective treatment early in the course of disease, the so-called window of opportunity, to avoid long term sequelae, such as joint damage. To accomplish this goal, it is essential to know beforehand who is going to respond well to MTX, so MTX monotherapy can be started in patients predicted to respond well, whereas other drugs, such as biologicals, could be prescribed to patients predicted to be non-responders.

In addition, MTX adverse effects, such as MTX intolerance, occur frequently and can lead to non-compliance, thus hindering its efficacy. Therefore, to avoid inefficacy of an otherwise efficacious drug, the physician should timely be aware of these adverse events.

## Objectives

The aim of this study was to identify predictors for MTX efficacy and adverse events.

## Methods

A systematic literature search was performed in PubMed, Embase and The Cochrane Library, and 1,331 articles were identified. These were selected based on their relevance, and critically appraised according to pre-defined criteria. Predictors for MTX efficacy and adverse events were tabulated.

## Results

Twenty articles were selected. For MTX efficacy, some interesting candidate predictors were found, such as antinuclear antibody positivity, the childhood health assessment questionnaire score, the myeloid-related protein 8/14 level, long-chain MTX polyglutamates, bilateral wrist involvement and some single nucleotide polymorphisms (SNPs) in the adenosine triphosphate binding cassette and solute carrier transporter gene families. For MTX adverse events, potential predictors were alanine aminotransferase and thrombocyte level and two SNPs in the γ-glutamyl hydrolase and methylenetetrahydrofolate reductase genes. Validation of most predictors was still lacking.

## Conclusion

Interesting candidate predictors were found, especially for MTX efficacy. However, most of these have not yet been validated in independent cohorts. The results of some candidate predictors were quite variable in different cohorts, highlighting the difficulty to properly validate those, due to heterogeneity between studies. A clinically relevant way to validate these predictors is by means of a clinical prediction model.

## Disclosure of interest

None declared.

